# The PERK–GADD45A axis is a key driver of hepatic stellate cell activation

**DOI:** 10.1097/HC9.0000000000000980

**Published:** 2026-06-19

**Authors:** Nipuni Barupala, Jagannath Misra, Emely Bibian, Reese Baxter, Vandana Singh, Noah Xique, Ethan D. Goins, Alex Jackson, Scott M. Ebert, Christopher M. Adams, Jessica L. Maiers

**Affiliations:** 1 Department of Anatomy, Cell Biology, and Physiology, Indiana University School of Medicine, Indianapolis, Indiana, USA; 2 Department of Biochemistry, Molecular Biology and Pharmacology, Indiana University School of Medicine, Indianapolis, Indiana, USA; 3 Department of Medicine, Indiana University School of Medicine, Indianapolis, Indiana, USA; 4 Indiana University Simon Comprehensive Cancer Center, Indiana University School of Medicine, Indianapolis, Indiana, USA; 5 Division of Endocrinology, Diabetes, Metabolism and Nutrition, Department of Medicine, Mayo Clinic, Rochester, Minnesota, USA

**Keywords:** endoplasmic reticulum stress, HSC, liver fibrosis, TGFβ, unfolded protein response

## Abstract

**Background:**

Hepatic stellate cells (HSCs) play a pivotal role in driving fibrosis during chronic liver injury. HSCs produce vast amounts of fibrotic proteins, causing endoplasmic reticulum (ER) stress and initiating the unfolded protein response (UPR). While the UPR is important for fibrogenesis, how signaling through UPR transducer Protein Kinase R-like ER Kinase (PERK) and its effectors impact HSC activation and fibrogenesis is unclear. Here, we sought to uncover the role of PERK and its effector GADD45A in liver fibrosis.

**Methods:**

PERK–GADD45A signaling was assessed in primary and immortalized HSCs treated with TGFβ, and mouse models of fibrosis. Genetic and pharmacological disruption of PERK or GADD45A was used to assess the role of PERK or GADD45A in HSC activation, proliferation, and fibrogenesis. HSC-specific *Gadd45a*-null mice were utilized to investigate the role of GADD45A on CCl_4_-induced fibrosis.

**Results:**

We found that TGFβ-induction of collagen I drives activation of PERK signaling in HSCs. Furthermore, loss or inhibition of PERK limits long-term HSC activation as illustrated by reduced levels of collagen I and fibronectin, impaired collagen I deposition, and reduced cell proliferation in vitro. Next, we show that PERK signaling induces expression of GADD45A during HSC activation, and loss of GADD45A disrupts HSC activation and expression of proliferation and cell-cycle-associated genes in immortalized and primary HSCs. Finally, HSC-specific GADD4Aa loss limits CCl_4_-driven fibrogenesis in vivo.

**Conclusion:**

PERK signaling is critical for HSC activation, and loss of the PERK downstream effector GADD45A limits fibrosis progression. Disruption of HSC activation and proliferation, coupled with dysregulation of cell-cycle-associated genes upon PERK or GADD45A loss, suggests that PERK–GADD45A signaling impacts multiple facets of HSC pathophysiology.

## INTRODUCTION

Chronic liver diseases cause over 1 million deaths annually worldwide, with cirrhosis and hepatocellular carcinoma (HCC) being late-stage complications that contribute to nearly 4% of global mortality.^1^ As chronic liver disease progresses, persistent injury drives fibrosis, which is characterized by the destruction and replacement of hepatocytes with extracellular matrix (ECM).^2^ Fibrosis can reverse if the injury is removed, but chronic insult can lead to cirrhosis and liver failure. Despite the rising challenge in prevention and treatment strategies, no approved therapeutics directly target fibrosis to date.^3^

HSCs comprise about 1.4% of the total volume of the liver and constitute 10% of all resident liver cells. Quiescent in nature, during liver injury, HSCs respond to a variety of soluble mediators and transdifferentiate into activated, highly proliferative, and ECM-producing myofibroblasts.^4^^,^^5^ This activation process exerts a heavy bioenergetic toll on HSCs and requires broad metabolic reprogramming to promote fibrosis.^6^ These processes include proliferation, migration, and production and secretion of fibrogenic proteins such as collagen I. Increased secretory load leads to the accumulation of unfolded or misfolded proteins in the endoplasmic reticulum (ER), thereby causing ER stress and initiation of the unfolded protein response (UPR) in HSCs.^7^^,^^8^ HSCs, rich in ER components and secretory proteins, are sensitive to disruptions of ER homeostasis, which can induce apoptosis if ER stress is not resolved.^9^^–^^11^ Induction of HSC apoptosis is associated with fibrosis resolution; thus, targeting UPR signaling could serve as a potential anti-fibrotic strategy across multiple types of liver disease.

The UPR is implicated in chronic liver disease and fibrosis.^12^^,^^13^ Treatment of HSCs with the pro-fibrotic cytokine TGFβ is sufficient to induce ER stress in HSCs, leading to signaling cascades downstream of ER stress sensors inositol-requiring enzyme 1α (IRE1α), activating transcription factor 6 alpha (ATF6α), and Protein Kinase R-like ER Kinase (PERK).^14^^–^^16^ Inhibition of IRE1α or ATF6α blocks TGFβ-induced myofibroblast activation, induces HSC apoptosis, reduces fibrosis in liver and skin models, and reverses fibrotic phenotypes in systemic sclerosis patients.^15^^,^^16^ These data suggest a feed-forward mechanism where UPR signaling is required to facilitate prolonged HSC activation and fibrogenesis downstream of TGFβ. This model is supported by HSC-specific deletion of ATF6α or pharmacological inhibition of IRE1α, reducing liver fibrosis in mice.^17^ While roles for IRE1α and ATF6α in HSC-driven fibrosis are well established, the contribution of PERK remains less understood. Canonically, PERK activation reduces global translation while selectively promoting translation of stress-responsive mRNAs, including ATF4.^18^ PERK phosphorylation is elevated in advanced fibrosis, and chemical induction of the UPR induced PERK signaling and HSC activation through an ATF4-independent pathway; however, the impact of pathophysiological drivers of HSC activation on PERK signaling remains unclear.^8^ ATF4 is a transcription factor that acts as a heterodimer with other transcription factors such as C/EBPβ (CCAAT/enhancer-binding protein beta) to promote expression of stress-responsive genes such as *Gadd45a.* ^19^^,^^20^ Interestingly, TGFβ induces both expression and phosphorylation of C/EBPβ through the IRE1α pathway, while ATF6α also induces c/EBPβ expression.^14^^,^^17^ This indicates that, similar to muscle atrophy, GADD45A may have a role in fibrogenesis. This led us to investigate the HSC-specific role of PERK and GADD45A signaling in driving liver fibrosis.

## METHODS

### Cell culture and treatment regimens

Human embryonic kidney cells with SV40 T antigen (HEK293T) were purchased from ATCC (#CRL-3216). Cells were grown in Dulbecco’s Modified Eagle Medium media (DMEM, Gibco 11965-092) supplemented with 10% (v/v) fetal bovine serum (FBS, Biowest S1480), and 100 units/mL penicillin and 100 μg/mL streptomycin (Cytiva, #SV30010). Human immortalized hepatic stellate cells (HSCs), LX-2 cells (a gift from Scott Friedman at Mt Sinai School of Medicine, NY, USA)^21^ were cultured at 37 °C and 5% CO_2_ in DMEM with 10% FBS Biowest S1480), and 1% Antibiotic/Antimycotic (ThermoFisher 15-140-122). Treatment doses and times for TGFβ (R&D Systems, #240-B), GSK2656157 (Selleckchem #7033) and 4µ8c (Selleckchem S7272), are described in the results and figure legends.

### Construction of cell lines

Cell lines expressing a non-targeting shRNA (shNT) or shRNAs targeting PERK or Col1a1 were generated via lentiviral transduction. HEK293T cells were transfected with lentiviral constructs with VSVG and P8.91 packaging plasmids using Effectene (Qiagen Cat. #301425) following the manufacturer’s instructions.^14^ Virus-containing supernatant was collected over a 48–72 hours period, spun down at 1500 rpm and filtered. LX-2 cells were transduced with virus-containing media supplemented with 10 µg/mL Polybrene (Millipore, Cat. #TR-1003-G) for 24 hours, after which the media were replaced with fresh growth medium. At 72 hours post-transduction, the media were switched to growth medium containing 1 µg/mL puromycin (Alfa Aesar, #J67236.8) for selection.

### In vivo experiments

8-10 week old male and female c57BL/6J mice receieved intraperitoneal (IP) injection of CCl_4_ (0.5 μL/mg dissolved in corn oil (CO), 0.08 mL/mouse) twice weekly for 6 weeks or CO as a control. A second cohort of 8-10 week old c/57Bl6 mice was fed a high-fat diet (Research Diets: D12109C; 20% calories from protein, 40% calories from fat, 40% calories from carbohydrate, 1.25% cholesterol, and 0.5% sodium cholate by weight) or a matched chow diet (D12102C) for 8 weeks. Both male and female mice were used for these studies. For conditional deletion of *Gadd45a* from HSCs, *Gadd45a* ^fl/fl^ mice were generated at the University of Iowa Genome Editing Core Facility using CRISPR/Cas9-based gene editing to insert LoxP sites between exons 1 and 2 and between exons 3 and 4 of the mouse *Gadd45a* gene in mice with a c57BL/6J background. Homozygous *Gadd45a* ^fl/fl^ mice were bred with *Pdgfrb* ^CreERT2^ expressing mice to yield *Gadd45a* ^fl/fl^ or *Gadd45a* ^fl/fl^ *Pdgfrb* ^CreERT2^ mice. The breeding colony was established at the Indiana University School of Medicine animal facility. Animal procedures were performed according to protocol approved by Indiana University Institutional Animal Care and Use Committee (Protocol number A24006). Animals were housed in individually ventilated cages under a 12-hour light/12-hour dark cycle with ad libitum access to food and water. Sex and age-matched littermates were injected with tamoxifen via IP injection (75 mg/kg, 5 consecutive days) to yield *Gadd45a* ^fl/fl^ or *Gadd45a* ^HSCΔ/Δ^ mice. These mice received CCl_4_ 2× weekly for 6 weeks by IP injection (0.5 μL/mg dissolved in corn oil, 0.08 mL/mouse), with paired littermate mice receiving CO as a control. Upon completion of the experiment, mice were euthanized, and livers were harvested for subsequent analyses. For immunoblotting, livers were lysed in a modified RIPA buffer (20 mM HEPES pH 7.5, 0.5% DOC, 0.2% SDS, 150 mM NaCl, 2 mM EDTA, 1% Triton plus protease inhibitors) and centrifuged to remove insoluble debris. For qPCR, mRNA was extracted from the whole liver using a Qiagen RNeasy kit and reverse transcribed.

### Statistical analysis

Data is presented as mean ± standard deviation. Statistical analyses were performed to confirm significance. For experiments with 2 conditions, the Student *t* test was used. For experiments with 3 or more conditions, either one-way ANOVA or two-way ANOVA followed by post hoc analyses were utilized where appropriate. Details are included in the figure legends for each panel.

Other experimental details are described in the Supplemental Materials and Methods, http://links.lww.com/HC9/C363.

## RESULTS

### TGFβ promotes PERK activation in HSCs through the induction of collagen I

**FIGURE 1:**
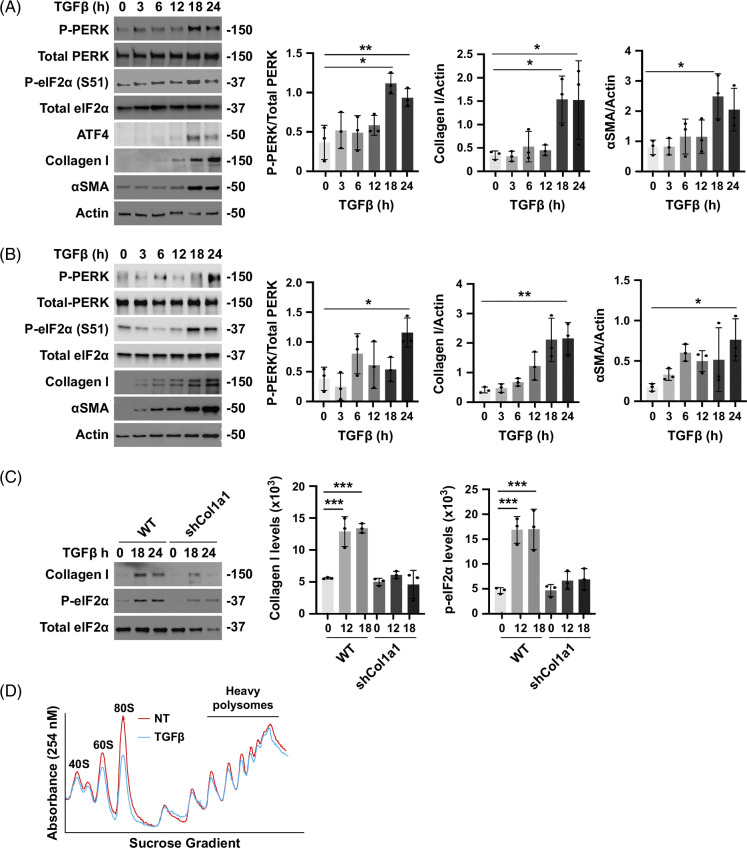
TGFβ promotes PERK activation in HSCs through the induction of collagen I. Primary mouse HSCs (A) or LX-2 cells (B) were treated with 2 ng/mL or 5 ng/mL of TGFβ, respectively, for 0, 3, 6, 12, 18, or 24 hours; followed by immunoblotting with the indicated antibodies. (C) LX-2 cells expressing shRNA targeting Col1a1 or a non-targeting control (shNT) were treated with TGFβ (5 ng/mL) for 0, 18, and 24 hours. Lysates were harvested and immunoblotted with the indicated antibodies. (D) LX-2 cells were treated with TGFβ (5 ng/mL) or vehicle for 18 hours and subjected to polysome profiling. Statistical significance was denoted by *; *p<0.05, **p<0.01, ***p<0.001, and ****p<0.0001 by one-way ANOVA (A and B) and two-way ANOVA (C). Error bars indicate mean ± SD (n=3). Abbreviations: ANOVA, analysis of variance; ATF4, activating transcription factor 4; Col1a1, collagen type I alpha 1 chain; eIF2α, eukaryotic initiation factor 2α; HSCs, hepatic stellate cells; PERK, protein kinase R-like ER kinase; SD, standard deviation; shNT, short hairpin RNA non-targeting control; shRNA, short hairpin RNA; TGFβ, transforming growth factor beta; WT, wild type; αSMA, alpha smooth muscle actin.

Prior studies showed that PERK activation, measured by autophosphorylation (P-PERK), is elevated in HSCs in response to tunicamycin and thapsigargin, chemical drivers of ER stress.^8^ To determine whether fibrogenic stimuli also induce PERK activation, we isolated HSCs from wild-type mice (mHSCs) and treated them with TGFβ (2 ng/mL) for increasing periods of time. Immunoblot analysis showed that P-PERK increased in response to TGFβ in a time-dependent manner, peaking at 18 hours post-TGFβ treatment and remaining elevated at 24 hours (Figure 1A). Levels of eIF2α phosphorylation at S51 (P-eIF2α) and ATF4 protein levels, downstream targets of PERK, followed the pattern of PERK activation. Corroboratively, ATF4 protein levels were robustly elevated in response to TGFβ at 18 hours (Figure 1A). Collagen I and αSMA, markers of activated HSCs, also showed a significant increase in response to TGFβ at 18 and 24 hours. We confirmed these findings in immortalized human HSCs (LX-2 cells), observing a similar pattern of PERK activation, along with increasing levels of collagen I and αSMA following TGFβ treatment, albeit with a slight delay, peaking around 24 hours (Figure 1B). We previously reported that TGFβ-mediated activation of IRE1α was reduced in cells expressing an shRNA targeting collagen I^14^; therefore, we explored whether loss of collagen I also limited TGFβ-mediated activation of PERK. LX-2 cells stably expressing shRNA targeting *Col1a1* (shCol1a1) were stimulated with TGFβ for 0, 18, or 24 hours, and immunoblotting revealed a significant reduction in P-eIF2α following TGFβ treatment levels in shCol1a1 cells compared with control cells (shNT) (Figure 1C). P-PERK and ATF4 levels were also reduced in shCol1a1 cells at 24 hours (Supplemental Figure S1, http://links.lww.com/HC9/C365), suggesting that enhanced collagen I synthesis during HSC activation triggers PERK and ISR activation.

P-eIF2α is canonically associated with reduced bulk translation and induction of translational control.^22^^–^^24^An important analysis of translational control is polysome profiling, which measures the occupancy of ribosomes on mRNA.^22^^–^^25^ We performed polysome profiling following 18 hours of TGFβ treatment to provide insight into translation dynamics during times where PERK activation is observed. Surprisingly, we did not observe robust changes in heavy polysomes (peaks further along the *x*-axis) between TGFβ or vehicle-treated samples (Figure 1D), suggesting that activation of PERK in response to TGFβ may not trigger canonical translational regulation as was recently reported.^26^ Together, these findings showcase that PERK signaling is increased in HSCs in response to TGFβ induction of collagen I, without concurrent robust changes in global protein synthesis.

### PERK depletion limits HSC activation

**FIGURE 2:**
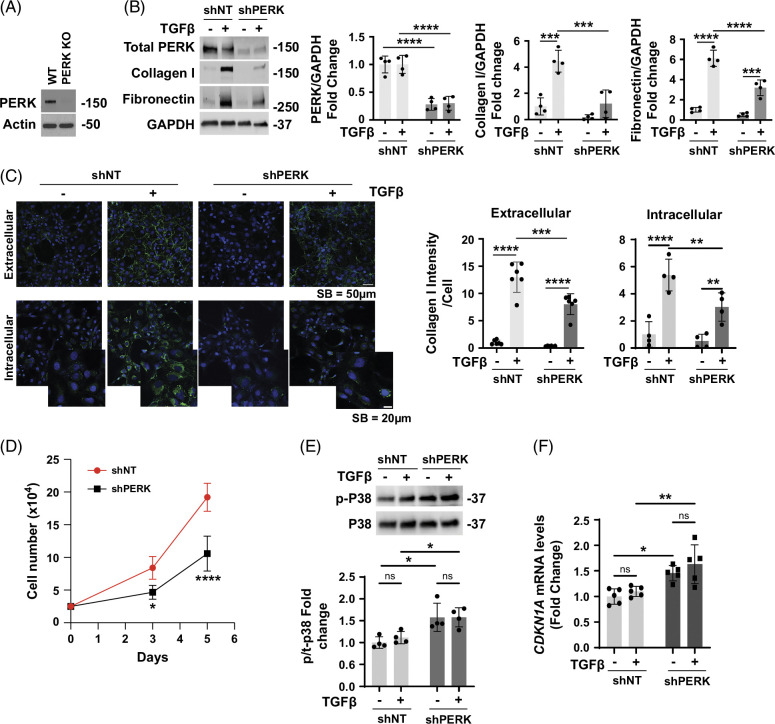
PERK depletion reduces collagen I deposition, limits HSC activation and proliferation. (A) CRISPR-Cas9-mediated PERK deletion in LX-2 cells was confirmed by immunoblotting. (B) PERK expression was knocked down using lentiviral-delivered shRNA, with shNT cells serving as controls. shPERK and shNT cells were treated with TGFβ (5 ng/mL) for 24 hours, followed by immunoblot with indicated antibodies (n=4). (C) shNT and shPERK cells were treated with TGFβ (5 ng/mL) for 48 (top panels) or 24 hours (bottom panels), fixed, and immunostained for collagen I. The top panels show collagen I deposition by staining non-permeabilized cells (SB=50 µm), and the bottom images show intracellular collagen I in permeablized cells (SB=20 µm). In all, 5–6 images were taken per condition and quantified for collagen I intensity (n=4–6). (D) shNT and shPERK cells were seeded and counted over 7 days to assess cell proliferation (n=3). (E) shNT and shPERK cells treated with either vehicle or TGFβ (5 ng/mL) for 24 hours, followed by immunoblot with indicated antibodies (n=4). (F) shNT and shPERK cells treated with either vehicle or TGFβ (5 ng/mL) for 24 hours and followed by qPCR analyses (n=5). Statistical significance was denoted by *; *p<0.05, **p<0.01, ***p<0.001, and ****p<0.0001 by two-way ANOVA. Error bars indicate mean ± SD (n=3 if not noted otherwise). Abbreviations: ANOVA, analysis of variance; CRISPR, clustered regularly interspaced short palindromic repeats; HSC, hepatic stellate cell; PERK, protein kinase R-like ER kinase; qPCR, quantitative polymerase chain reaction; SB, scale bar; SD, standard deviation; shNT, short hairpin RNA non-targeting control; shPERK, short hairpin RNA targeting PERK; shRNA, short hairpin RNA; TGFβ, transforming growth factor beta; WT, wild type; αSMA, alpha smooth muscle actin.

To assess the role of PERK in HSC activation, we first generated a genetic knockout (KO) of PERK in LX-2 cells using CRISPR-Cas9 (Figure 2A); however, clonal selection of these cells was unsuccessful as PERK-KO cells failed to proliferate. To overcome this, we infected LX-2 cells with an shRNA targeting PERK to generate cells with stable knockdown of PERK (shPERK). We confirmed PERK reduction by immunoblot analysis and qPCR (Figure 2B and Supplemental Figure S2A, http://links.lww.com/HC9/C366 left panel, respectively). PERK knockdown significantly reduced TGFβ induction of collagen I and fibronectin protein levels compared with control cells (Figure 2B). Interestingly, the impact of PERK loss on *COL1A1* or *FN1* mRNA levels was less pronounced. While PERK loss decreased the induction of *COL1* and *FN1*, these genes were still significantly induced by TGFβ (Supplemental Figure S2A, http://links.lww.com/HC9/C366 middle panels). In contrast, induction of *ACTA2* was ablated in shPERK cells. Supplemental Figure S2A, http://links.lww.com/HC9/C366, right panel), suggesting that PERK depletion limits HSC activation. To support our genetic approach, we inhibited PERK pharmacologically using GSK2656157.^27^ Pretreatment of LX-2 cells with 1 µM or 2 µM GSK1656157 inhibited PERK phosphorylation and limits ATF4 induction by TGFβ, thus we used 2 µM GSK2656157 for additional experiments (Supplemental Figure S2B, http://links.lww.com/HC9/C366). Pretreatment of LX-2 cells or mHSCs with GSK2656157 significantly blunted TGFβ-induced collagen I protein levels (Supplemental Figures S2C, D, http://links.lww.com/HC9/C366). Interestingly, PERK inhibition did not significantly change *COL1A1* mRNA levels in LX-2 cells, though inhibition of IRE1α reduced *COL1A1* levels consistent with previous reports (Supplemental Figure S2E, http://links.lww.com/HC9/C366).^14^ As we observed a greater impact on collagen I protein compared with mRNA, we asked whether disrupting PERK altered SMAD2/3 signaling. We assessed SMAD3 phosphorylation in shNT and shPERK cells in response to TGFβ and found that PERK depletion did not significantly affect SMAD3 phosphorylation after 1 or 4 hours; however, SMAD3 phosphorylation was reduced after prolonged TGFβ exposure (12 or 24 h, Supplemental Figures S2F, G, http://links.lww.com/HC9/C366), aligning with decreased collagen I mRNA observed in shPERK cells. To further validate our findings, we transiently reduced PERK or ATF4 by siRNA in LX-2 cells, followed by TGFβ treatment. In agreement with the previous results, depletion of either PERK or ATF4 led to a significant reduction of collagen I protein following TGFβ treatment (Supplemental Figure S3A, http://links.lww.com/HC9/C367). Overall, these results demonstrate the importance of PERK in HSC activation, with PERK and UPR activation as a consequence of heightened synthesis of matrix proteins during HSC activation, and PERK and UPR activation required to sustain the HSC activation.

### PERK inhibition reduces collagen I deposition and HSC proliferation

The impact of PERK loss on TGFβ-induction of fibrogenic proteins led us to assess whether PERK depletion impacted functional readouts of HSC activation: extracellular collagen I deposition and proliferation. Secretion and deposition of collagen I is a critical step in fibrogenesis and a hallmark of fibrosis. We treated shPERK or shNT cells with TGFβ for 24 or 48 hours, fixed the cells, and either permeabilized (24 h samples) to assess total collagen levels or left the cells unpermeabilized (48 h samples) to preferentially assess extracellular collagen I. Immunofluorescence revealed a significant reduction in both total and deposited collagen I in shPERK cells in response to TGFβ compared with shNT cells (Figure 2C); supported by experiments where PERK inhibition reduced collagen I deposition in both LX-2 cells and mHSCs (Supplemental Figure S3B, http://links.lww.com/HC9/C367). Next, we explored whether PERK loss impaired HSC proliferation, particularly as PERK deletion by CRISPR-Cas9 disrupted cell growth. We plated equal numbers of shNT and shPERK cells (day 0) and counted cells at days 3 and 5. shPERK cells exhibited significantly reduced proliferation compared with control cells (Figure 2D). GSK2656157 similarly reduced LX-2 cell proliferation over time, indicating that PERK is pivotal for HSC proliferation (Supplemental Figure S3C, http://links.lww.com/HC9/C367). Cell viability, measured using trypan blue during the cell counts, did not show significant changes between control and shPERK cells, suggesting the effects were not due to increased cell death (data not shown). Next, we sought to understand how PERK depletion affects HSC proliferation. Immunoblot showed that PERK loss led to increased levels of p-p38, but not p-ERK (Figure 2E, data not shown). As p38 MAPK signaling through p21 can promote G1/S cell-cycle arrest, leading to growth inhibition, we measured p38 phosphorylation and *CDKN1A* (p21) levels in control and shPERK cells following TGFβ treatment.^28^ Basal *CDKN1A* expression was significantly elevated in shPERK cells, which further increased upon TGFβ treatment (Figure 2F), indicating that PERK loss restricts cell proliferation through a p38–p21 pathway.

Our results show that PERK loss reduces collagen I expression and deposition, and impairs proliferation. These findings suggest that PERK is not merely a stress sensor but is required to sustain the activated, collagen-producing state of HSCs. Given these defects, we assessed UPR activation in response to TGFβ, observing TGFβ induction of all 3 UPR branches, PERK, IRE1α, and ATF6α, consistent with recent reports.^29^ However, in shPERK cells, not only is PERK downstream signaling (ATF4) suppressed, but levels of IRE1α (sXBP1) and nuclear ATF6α (nATF6α) are also reduced, indicating that global UPR activation is compromised (Supplemental Figure S3D, http://links.lww.com/HC9/C367). These findings are consistent with previous reports showing roles for PERK in IRE1α expression and XBP1 mRNA splicing, and efficient ATF6α activation.^30^^,^^31^ We also analyzed the impact of PERK loss on ER-associated degradation (ERAD), a mechanism that aims to relieve ER stress during secretory stress. Of note, both *HERPUD1* and *HRD1* were significantly upregulated in PERK-depleted cells, implying ERAD-associated gene induction in the absence of PERK (Supplemental Figure S3E, http://links.lww.com/HC9/C367). These data suggest that loss of PERK disrupts coordinated UPR signaling, leading to defective stress adaptation and, consequently, impaired HSC activation, collagen production, and proliferation.

### PERK mediates TGFβ-induction of GADD45A

**FIGURE 3:**
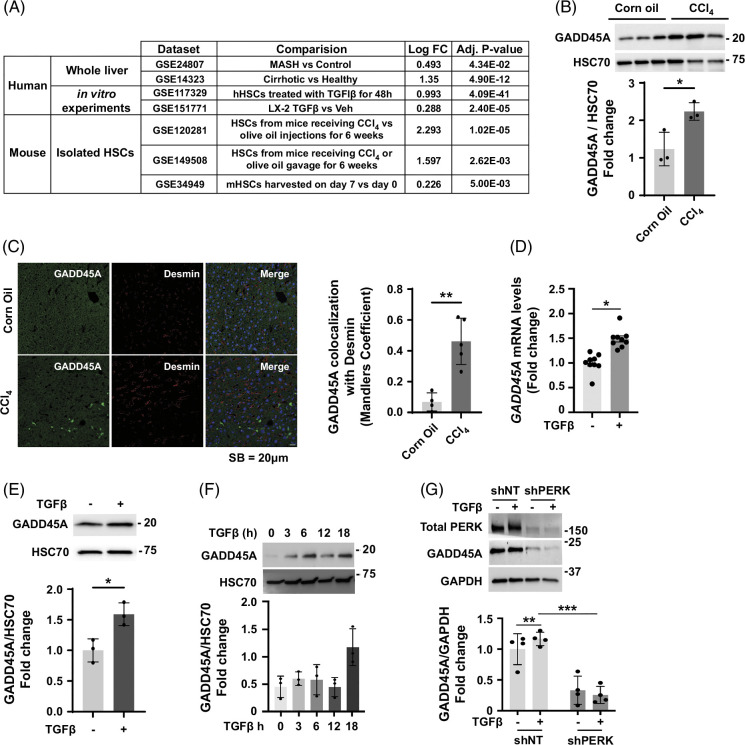
PERK mediates TGFβ-induction of GADD45A.TGFβ. (A) In silico analysis of GADD45A expression in liver tissue and HSCs using publicly available datasets. (B, C) Male WT mice received either corn oil (CO) or CCl_4_ twice weekly for 6 weeks. Whole liver lysates were analyzed by immunoblot with indicated antibodies (n=3) (B), and liver tissue was analyzed by immunofluorescence (n=4) (C). For (C), desmin was used as an HSC marker, and semi-quantitation of double-positive cells was performed (SB=20 µm). (D, E) Primary human HSCs were treated with TGFβ (2 ng/mL) for 24 hours, and GADD45A levels were quantified by qPCR (n=8) (D) and immunoblot (n=3) (E). (F) mHSCs isolated from WT mice were treated with TGFβ (2 ng/mL) for 0, 3, 6, 12, or 18 hours, followed by immunoblot with indicated antibodies (n=3). (G) shNT and shPERK cells were treated with TGFβ (5 ng/mL) for 24 hours, followed by immunoblot with indicated antibodies (n=4). Statistical significance was denoted by *; *p<0.05, **p<0.01, and ***p<0.001 by *t* test (B, C, D, and E). One-way ANOVA (F), and two-way ANOVA (G). Error bars indicate mean ± SD (n=3 if not noted otherwise). Abbreviations: ANOVA, analysis of variance; CCl_4_, carbon tetrachloride; CO, corn oil; GADD45A, growth arrest and DNA damage inducible α; HSCs, hepatic stellate cells; MASH, metabolic dysfunction–associated steatohepatitis; mHSCs, mouse hepatic stellate cells; PERK, protein kinase R-like ER kinase; qPCR, quantitative polymerase chain reaction; SB, scale bar; SD, standard deviation; shNT, short hairpin RNA non-targeting control; shPERK, short hairpin RNA targeting PERK; TGFβ, transforming growth factor beta; WT, wild type.

The impact of PERK loss or inhibition on HSC activation and proliferation led us to question how PERK/ATF4 signaling promotes fibrogenesis. As ATF4 knockdown reduced TGFβ-induction of collagen I, we hypothesized that a mediator downstream of ATF4 was required for HSC activation. ATF4 is known to function as a heterodimer with various b-ZIP transcription factors, including C/EBPβ and others,^32^ to regulate gene expression under stress conditions. The transcription factor c/EBPβ was found to be regulated through the UPR during HSC activation by TGFβ,^14^^,^^17^ and known to heterodimerize with ATF4 to transcriptionally regulate stress-inducible gene GADD45A.^32^^,^^33^ Through in silico analysis of publicly available datasets, we observed elevated GADD45A expression in patients with metabolic dysfunction–associated steatohepatitis (MASH) or cirrhosis compared with controls and in primary and immortalized HSCs (Figure 3A). We confirmed this finding in a mouse model of fibrosis, where GADD45A increased in liver tissue following 6 weeks of biweekly CCl_4_ injection of corn oil (CO) as a control (Figure 3B). We additionally performed immunofluorescence staining for GADD45A on liver tissue from these mice, alongside the HSC marker Desmin. We found that GADD45A increased in livers from CCl_4_-treated mice, and GADD45A staining colocalized with Desmin-positive cells (Figure 3C). While this increase was not exclusive to Desmin-positive cells, it supported a potential role for GADD45A in HSCs. We next treated primary human HSCs (hHSCs, ScienCell) with TGFβ for 24 hours and observed a significant increase in *GADD45A* mRNA levels (Figure 3D) and protein levels (Figure 3E). Similarly, TGFβ treatment of isolated mHSCs resulted in a time-dependent increase in GADD45A expression (Figure 3F), which paralleled the activation of PERK signaling (Figure 1A).

The increase of GADD45A in TGFβ-treated HSCs led us to investigate whether TGFβ induction of GADD45A is regulated via PERK signaling. shNT or shPERK cells were treated with TGFβ for 24 hours, and immunoblotting revealed that GADD45A levels were significantly reduced in shPERK cells (Figure 3G), while siRNA knockdown of PERK or ATF4 also reduced GADD45A levels following TGFβ treatment (Supplemental Figure S4A, http://links.lww.com/HC9/C368). Supporting this, pretreatment with the PERK inhibitor GSK2656157 significantly reduced TGFβ-induction of GADD45A mRNA and protein levels in LX-2 cells (Supplemental Figure S4B, C, http://links.lww.com/HC9/C368). This prompted us to examine the role of GADD45A in driving the profibrogenic response in vitro.

### Loss of GADD45A decreases expression and deposition of fibrogenic genes

**FIGURE 4:**
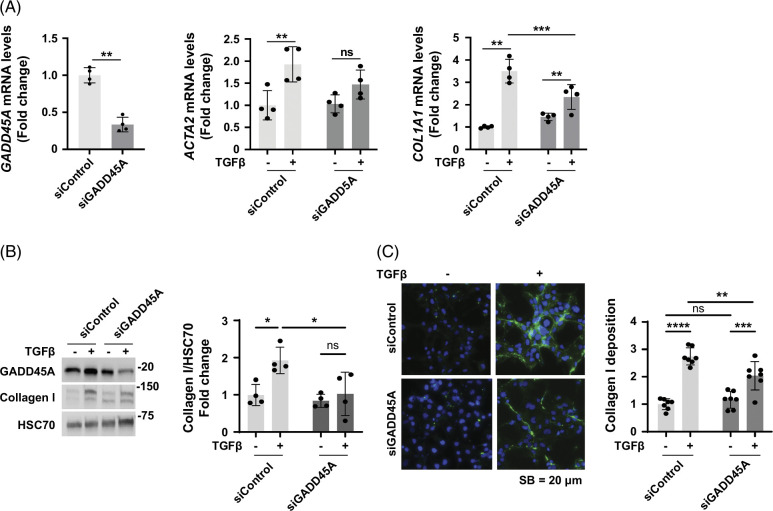
Loss of GADD45A decreases expression and deposition of fibrogenic genes. (A–C) LX-2 cells were transfected with either siRNA targeting GADD45A or scrambled siRNA (siControl), followed by TGFβ (5 ng/mL) treatment for 24 hours. (A) qPCR analyses were performed to measure *GADD45A*, *ACTA2*, and *COL1A1* (n=4). (B) Immunoblot analyses were carried out with the indicated antibodies (n=3). (C) Cells were fixed and immunostained to measure extracellular collagen I deposition. Abbreviations: ACTA2, actin alpha 2 smooth muscle; COL1A1, collagen type I alpha 1 chain; GADD45A, growth arrest and DNA damage inducible α; LX-2, human hepatic stellate cell line LX-2; qPCR, quantitative polymerase chain reaction; siControl, scrambled small interfering RNA control; siRNA, small interfering RNA; TGFβ, transforming growth factor beta.

**FIGURE 5:**
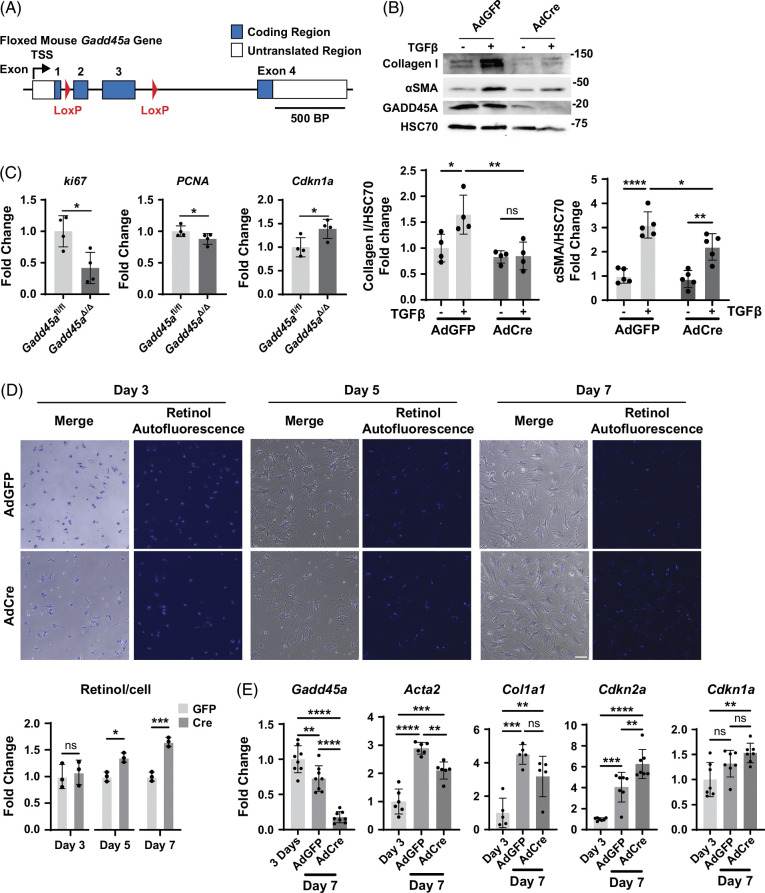
Gadd45a deletion from mHSCs limits HSC activation and proliferation. (A) Schematic showing the strategy to flox the *Gadd45a* gene in a mouse model. (B) mHSCs were isolated from *Gadd45a* ^fl/fl^ mice, checked for retinol expression, infected with AdCre or AdGFP for 24 hours, and then treated with TGFβ (2 ng/mL). The levels of GADD45A, collagen I, and αSMA were measured by immunoblotting (n=4, quantification below). (C) qPCR analyses of proliferation and cell-cycle markers 48 hours following adenoviral infection (N=4). (D) Isolated HSCs were infected with adGFP or adCre, followed by culturing for up to 7 days. Cells were imaged at 3, 5, and 7 days to assess retinol autofluorescence, analyzed as integrated density of the signal divided by cell number (SB=100 µm). (E) qPCR analyses of mHSCs following 3 days (adGFP) or 7 days (adGFP or adCre) of culture. Statistical significance was denoted by *; *p<0.05, **p<0.01, ***p<0.001, and ****p<0.0001 by *t* test (A left panel) and two-way ANOVA (A, B, C, E, and F). Error bars indicate mean ± SD (n=3 if not noted otherwise). Abbreviations: AdCre, adenovirus expressing Cre recombinase; AdGFP, adenovirus expressing green fluorescent protein; ANOVA, analysis of variance; GADD45A, growth arrest and DNA damage inducible α; HSCs, hepatic stellate cells; mHSCs, mouse hepatic stellate cells; qPCR, quantitative polymerase chain reaction; SB, scale bar; SD, standard deviation; TGFβ, transforming growth factor beta; αSMA, alpha smooth muscle actin.

To understand the role of GADD45A during HSC activation, we knocked down GADD45A in LX-2 cells using siRNA (Figure 4A). Silencing GADD45A significantly reduced the TGFβ-induced expression of fibrogenic genes, including αSMA/*ACTA2* and collagen I (Figure 4A). At the protein level, GADD45A knockdown also led to decreased collagen I levels (Figure 4B) and reduced extracellular deposition of collagen I (Figure 4C). Given these results, we next tested whether GADD45A plays a similar role in primary mHSCs. We isolated mHSCs from *Gadd45a* ^fl/fl^ mice where loxP sites were inserted between exons 1 and 2, and exons 3 and 4 (Figure 5A). *Gadd45a* ^fl/fl^ HSCs were infected with either AdCre to induce recombination or AdGFP as a control. After infection, cells were treated with TGFβ for 24 hours and harvested for immunoblotting. *Gadd45a* deletion markedly suppressed TGFβ-induced collagen I and αSMA expression (Figure 5B). qPCR analysis of mHSCs after 48 hours of infection also showed reduced expression of proliferation markers *ki67* and *Pcna*, and increased expression of *Cdkn1a/*p21 (Figure 5C). We next investigated the impact of *Gadd45a* loss on stiffness-induced HSC activation. mHSCs were isolated from *Gadd45a* ^fl/fl^ mice and infected with AdGFP or AdCre. After 3, 5, or 7 days of culture, cells were imaged for retinol droplets, which were quantified using ImageJ and normalized to cell number. The amount of retinol/cell was elevated in AdCre-infected cells compared with AdGFP controls, indicative of a more quiescent phenotype in the absence of *Gadd45a* (Figure 5D). Finally, mRNA was harvested at baseline (3 d), or 7 days post isolation, revealing that loss of *Gadd45a* led to significant reduction in *Acta2* and increased expression of *Cdkn2a/*p16 and *Cdkn1a*/p21 (Figure 5E). *Col1a1* was not significantly changed.

### GADD45A loss limits CCl_4_-mediated fibrogenesis

**FIGURE 6:**
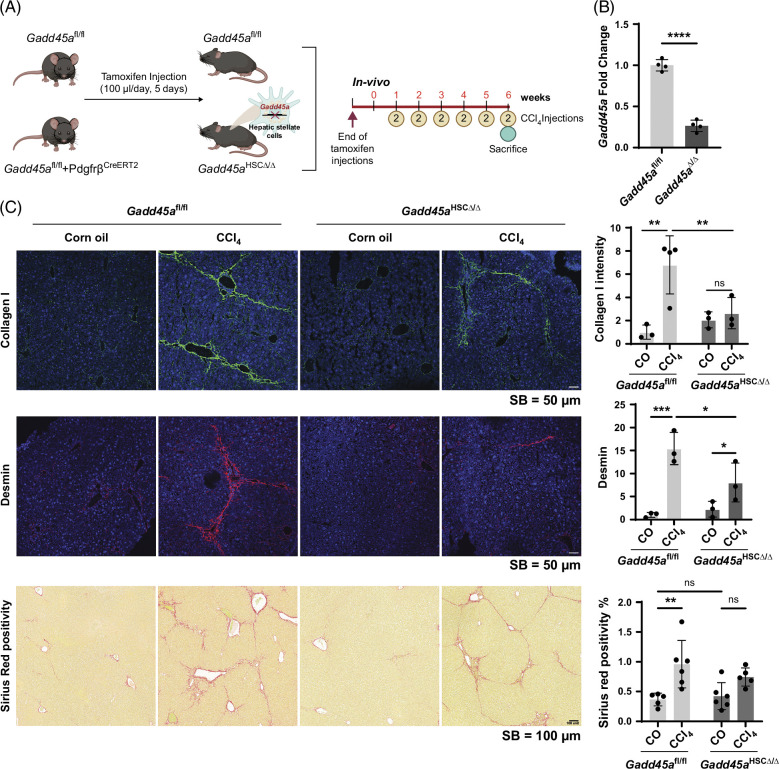
HSC-specific GADD45A deletion limits CCl_4_-mediated fibrogenesis. (A) *Gadd45a* ^*fl/fl*^ mice were crossed with *Pdgfrb* ^CreERT2^ mice to generate tamoxifen-inducible, HSC-specific *Gadd45a* knockout mice (*Gadd45a* ^HSCΔ/Δ^). Age-matched and sex-matched *Gadd45a* ^fl/fl^ and *Gadd45a* ^HSCΔ/Δ^ littermates received tamoxifen injections (75 mg/kg IP daily for 5 d) to induce Cre-mediated recombination. One week post-tamoxifen treatment, liver fibrosis was induced using intraperitoneal CCl_4_ injections (0.5 µL/mg, 0.08 mL/mouse) twice weekly for 6 weeks; corn oil (CO) injections served as the control treatment. The livers were harvested and formalin-fixed paraffin-embedded (PFFE). Diagram generated using Biorender https:// BioRender.com/8cxjonv (B) qPCR analysis confirmed that tamoxifen injection significantly reduced *Gadd45a* mRNA levels in the mouse liver (n=4). (C) Representative images of collagen I or desmin immunofluorescence, and Sirius Red staining of control and CCl_4_-treated liver sections from *Gadd45a* ^fl/fl^ and *Gadd45a* ^HSCΔ/Δ^ mice (n=3 and 5, respectively; SB 100 mM). Statistical significance was denoted by *; *p<0.05, **p<0.01, ***p<0.001, and ****p<0.0001 by *t* test (B) and two-way ANOVA (C, D). Error bars indicate mean ± SD (n=3 if not noted otherwise). ANOVA, analysis of variance; CCl_4_, carbon tetrachloride; CO, corn oil; FFPE, formalin-fixed paraffin-embedded; HSC, hepatic stellate cell; IP, intraperitoneal; qPCR, quantitative polymerase chain reaction; SB, scale bar; SD, standard deviation.

**FIGURE 7:**
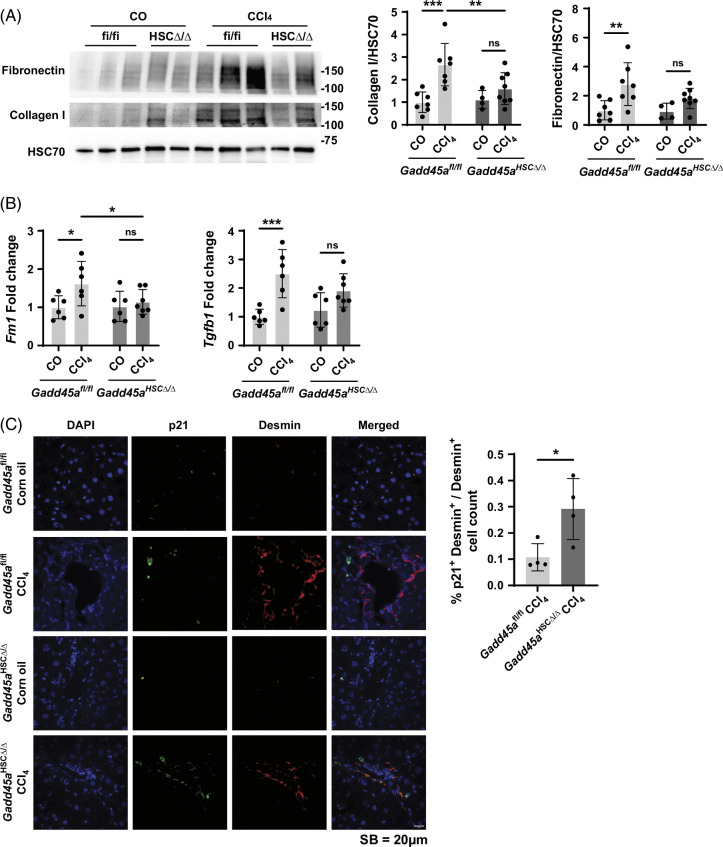
HSC-specific GADD45A deletion limits HSC activation markers and increases p21+Desmin+cells. (A) Immunoblot analyses of total liver lysate from control and CCl_4_-treated groups (n=4–8). (B) mRNA levels of fibronectin (*Fn1*) and transforming growth factor beta-1 (*Tgfb1*) were measured in control and *Gadd45a* ^HSCΔ/Δ^ mice injected with either vehicle or CCl_4_ (n=6–7). (C) Frozen tissue sections of *Gadd45a* ^fl/fl^ and *Gadd45a* ^HSCΔ/Δ^ mice injected with either CO or CCl_4_ were sectioned and immunostained for desmin and p21. ImageJ was utilized to count Desmin-positive and p21-positive cells. Statistical significance was denoted by *; *p<0.05, and **p<0.01 by *t* test (p21/Desmin) or two-way ANOVA. Error bars indicate mean ± SD (n=3 if not noted otherwise). Abbreviations: ANOVA, analysis of variance; CCl_4_, carbon tetrachloride; CO, corn oil; Fn1, fibronectin 1; HSC, hepatic stellate cell; ImageJ, Image Processing and Analysis in Java; mRNA, messenger RNA; p21, cyclin-dependent kinase inhibitor 1A; SD, standard deviation; Tgfb1, transforming growth factor beta-1.

Given the role of GADD45A in HSC activation in vitro, we investigated whether HSC-specific deletion could attenuate liver fibrosis in vivo. We crossed *Gadd45a* ^fl/fl^ mice with mice expressing a tamoxifen-inducible Cre recombinase under the control of the platelet-derived growth factor receptor β promoter (*Pdgfrb* ^CreERT2^) (Figure 6A). Age-matched and gender-matched littermates (G*add45a* ^fl/fl^ and G*add45a* ^fl/fl^+*Pdgfrb* ^CreERT2^) were injected with tamoxifen for 5 consecutive days, generating *Gadd45a* ^fl/fl^ and *Gadd45a* ^HSCΔ/Δ^ mice, respectively, confirming GADD45A deletion following tamoxifen injection by isolating mHSCs (Figure 6B). One week following the final tamoxifen injection, liver injury was induced using CCl_4_ injection twice a week for 6 weeks, with CO serving as a control. Analysis of liver sections revealed that CCl_4_ treatment increased Sirius Red staining, as well as desmin and collagen I immunofluorescence in *Gadd45a* ^fl/fl^ mice, whereas these effects were markedly reduced in *Gadd45a* ^HSCΔ/Δ^ mice (Figure 6C). Immunoblotting of liver tissue revealed significantly reduced expression of collagen I and fibronectin in *Gadd45a* ^HSCΔ/Δ^ mice following CCl_4_ injury, indicating reduced HSC activation (Figure 7A). We also found reduced mRNA levels of fibrosis-associated genes *Fn1* and *Tgfb1* in *Gadd45a* ^HSCΔ/Δ^ mice (Figure 7B). Next, we co-stained liver sections for Desmin and p21 to assess whether p21 levels in HSCs increased, similar to what was observed in isolated mHSCs (Figures 5C, E). *Gadd45a* ^HSCΔ/Δ^ had a higher number of p21+Desmin+ cells following CCl_4_ treatment compared with *Gadd45a* ^fl/fl^ mice (Figure 7C), further supported by IHC analysis of p21 and Desmin (Supplemental Figure S5, http://links.lww.com/HC9/C369). This suggests that loss of Gadd45a leads to increased p21 expression, pushing HSCs toward senescence and reducing their activation. Together, these data demonstrate that GADD45A regulates fibrogenic genes as a key driver of HSC activation and fibrogenesis in chronic liver injury.

## DISCUSSION

**FIGURE 8:**
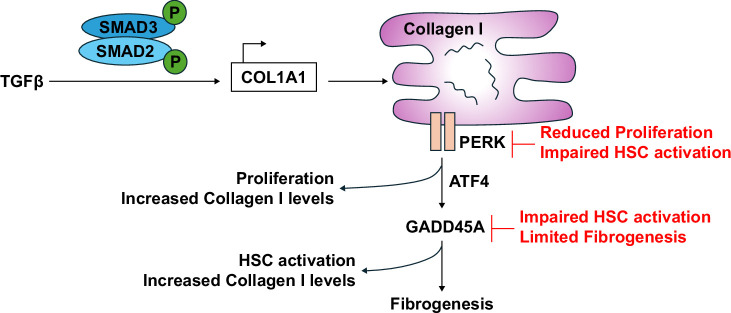
TGFβ induces collagen synthesis in HSCs, increasing ER protein-folding demand and activating the PERK–UPR pathway. PERK signaling, in turn, sustains HSC activation, proliferation, and collagen secretion partly through the induction of GADD45A. Loss of PERK or GADD45A disrupts fibrogenic gene expression and collagen deposition. During chronic liver injury, GADD45A deletion limits fibrotic responses, highlighting the PERK–GADD45A axis as a potential therapeutic target in liver fibrosis. Abbreviations: ER, endoplasmic reticulum; GADD45A, growth arrest and DNA damage inducible α; HSCs, hepatic stellate cells; PERK, protein kinase R-like ER kinase; TGFβ, transforming growth factor beta; UPR, unfolded protein response.

Upon liver injury, HSCs activate and increase protein production to promote repair; however, chronic injury drives persistent activation and fibrosis. The UPR supports this process by managing the heightened protein-folding and secretory demands of these cells,^6^ facilitating fibrogenic protein secretion and HSC viability.^14^^–^^17^ While stress responses support tissue repair, they also drive fibrosis, making them possible targets for treatment.^10^ Here, we investigated the fibrogenic role of pathological UPR signaling through PERK in HSC activation. We demonstrate for the first time that TGFβ activates PERK in HSCs (Figure 1 and Supplemental Figure 3A, http://links.lww.com/HC9/C367). Genetic or pharmacologic inhibition of PERK reduces TGFβ-induced fibrogenic protein levels, collagen deposition, and HSC proliferation (Figure 2). Together, these findings support a reactive model in which PERK activation is initially triggered by the increased protein-folding demands and functions adaptively to sustain collagen secretion and HSC activation. Mechanistically, PERK mediates the expression of GADD45A to promote fibrogenesis (Figure 3). Loss of GADD45A attenuates HSC activation and fibrogenesis in vitro and in vivo, indicating its essential role in this process (Figures 4–7), highlighting the PERK–GADD45A axis as a potential therapeutic target in liver fibrosis (Figure 8).

### PERK activation and signaling in HSCs

PERK activation during ER stress leads to eIF2α phosphorylation, global translation attenuation, and selective translation of stress-responsive genes to reduce ER protein load and promote cell survival.^18^^,^^34^ Although well characterized in hepatocytes, PERK’s role in HSCs activation is less clearly defined. Our findings demonstrate that TGFβ induces PERK activation in HSCs (Figures 1A, B). Interestingly, PERK signaling peaked around 18 hours, but remains elevated through 24 hours. This could be due to negative feedback loops aimed at limiting prolonged UPR activation or increased levels of chaperone proteins in response to TGFβ, as previously highlighted.^7^^,^^35^^,^^36^ We also observed that while P-eIF2α typically reduces global translation, polysome profiling did not reveal substantial repression of polysome occupancy following TGFβ treatment (Figure 1D), suggesting that PERK activation does not elicit a classical translational shutoff. Nevertheless, P-eIF2α and ATF4 induction indicate that PERK may function as a reactive sensor of collagen-induced ER stress, reprogramming gene expression to facilitate fibrogenesis.

Previous work showed elevated P-PERK in rodent models of liver fibrosis, as well as HSCs either isolated from fibrotic models or treated with chemical inducers of ER stress.^8^^,^^37^ These studies linked PERK signaling to fibrogenesis, but as chemical inducers of ER stress illicit non-physiological UPR signaling, our data showing a clear physiological induction of PERK signaling during HSC activation and a critical role for PERK in fibrogenesis is an important finding. PERK is also essential for cell growth. PERK knockout cells failed to proliferate (Figure 2A), and shPERK cells showed impaired proliferation accompanied by upregulation of p38 phosphorylation and p21 expression (Figures 2D–F). This may play a key role in vivo, limiting both ECM deposition and HSC number. We also observed that loss of PERK suppressed collagen I and fibronectin protein levels (Figure 2B) and reduced extracellular collagen deposition (Figure 2C) with limited changes in collagen I mRNA. This suggests that collagen I is disrupted at least in part through a post-transcriptional mechanism. Polysome profiling did not support a global decrease in protein translation; we postulated that PERK loss could influence protein degradation. There are 2 major degradative pathways associated with the UPR: ERAD and ER-lysosomal associated degradation (ERLAD), which includes ER-phagy.^38^^,^^39^ We previously showed an integral role for ER-phagy in maintaining collagen I homeostasis.^29^ This work focused on ATF6α, though we did observe that PERK inhibition limited TGFβ induction of ER-phagy receptor *CCPG1*. Furthermore, we show here that PERK loss led to increased levels of ERAD components. While preliminary, these data suggest that PERK could impact collagen I and HSC biology through regulating protein degradation.

ATF4 is a canonical effector of PERK signaling, and our data suggest that TGFβ induction of GADD45A in HSCs is ATF4-dependent. Recent studies have established ATF4 as an important regulator of fibrogenesis. First, ATF4 facilitates procollagen synthesis by enhancing serine-to-glycine conversion in lung fibroblasts, indirectly supporting extracellular matrix assembly in lung fibrosis.^40^ Second, ATF4 promotes HSC activation through EMT-related transcription, binding to enhancers of fibrogenic genes, suggesting a non-canonical, chromatin remodeling-based mechanism.^26^ Indeed, pharmacological inhibition of ATF4 or P-eIF2α markedly reduced fibrosis in this system. There is also evidence that PERK regulates HSC activation through ATF4-independent mechanisms. PERK activation promoted degradation of HNRNPA1, which led to increased SMAD2 levels and pro-fibrotic gene expression.^8^ These varying outcomes imply that PERK’s downstream pathways are context dependent, with PERK contributing to HSC activation and liver fibrosis via multiple mechanisms involving ATF4-dependent and ATF4-independent routes.

### Context-dependent roles of GADD45A in liver fibrosis

GADD45A is a key stress-responsive gene linked to skeletal muscle atrophy. In muscle, stressors such as fasting, denervation, and immobilization activate ATF4, which promotes atrophy by inducing *Gadd45a* expression.^19^^,^^20^ Consequently, GADD45A represses anabolic signaling pathways while activating catabolic mechanisms like autophagy and caspase-mediated proteolysis. Extending the relevance of this pathway to hepatic fibrosis, our results demonstrate that GADD45A is a downstream effector of TGFβ-induced PERK-P–eIF2α–ATF4 signaling in HSCs. GADD45A expression is upregulated in fibrotic livers from both human MASH and CCl_4_-treated mice (Figures 3A, B). TGFβ stimulation of primary human and mouse HSCs induces GADD45A mRNA and protein expression (Figures 3D–H), while knockdown of PERK or ATF4 blocks this effect (Figure 3G). Knockdown of GADD45A attenuates TGFβ-induced expression and deposition of fibrogenic markers such as collagen I and αSMA in vitro (Figure 4). Furthermore, HSC-specific deletion of *Gadd45a* in vivo significantly reduces liver fibrosis, HSC activation, and HSC proliferation following CCl_4_ injury (Figures 5, 6). These findings identify GADD45A as a shared downstream effector of stress-adaptive transcriptional programs that promote tissue remodeling in both muscle and liver fibrosis.^19^^,^^41^

Other studies indicated that GADD45A may play a protective role in liver injury and fibrosis. For instance, Hong et al^42^ reported that hepatic GADD45A expression was reduced in a 4-week CCl_4_-induced fibrosis model, and that loss of GADD45A *in vitro* promoted SMAD2/3 signaling in response to CCl_4_; however, their study did not include direct TGFβ stimulation of HSCs, a key driver of fibrogenesis, thereby limiting mechanistic insight into GADD45A’s role in HSC activation. In contrast, our study directly shows a fibrogenic role for GADD45A through the induction of collagen I and other fibrogenic markers. Similarly, Tanaka et al^43^ reported a hepatoprotective function for GADD45A in a MCD-induced liver injury model using *Gadd45a* KO mice, focusing primarily on hepatocyte stress responses without assessing its role in HSCs. Differential roles for GADD45A across hepatic cell types is not surprising, as GADD45A expression is not specific to HSCs, and likely plays important roles in other liver cell types, including endothelial cells and immune cells. Indeed, our data indicate that GADD45A is not induced in high-fat diet (HFD) models (Supplemental Figure S6, http://links.lww.com/HC9/C370) but is upregulated in fibrotic models such as CCl_4_ treatment (Figure 3B). Together, these differences underscore the model-dependent and context-dependent roles of GADD45A and highlight a previously unrecognized pro-fibrotic function for GADD45A in HSCs. We show that HSC-specific loss of GADD45A leads to increased p21-positive HSCs following CCl_4_ injection, associated with an anti-fibrotic phenotype. p21 is a cell-cycle inhibitor and driver of senescence regulated through p53, and both p53 and p38 MAPK are implicated in stress-induced senescence in HSCs. It is biologically plausible that GADD45A modulates this network by influencing p21 expression and limiting HSC proliferation and activation. Functionally, HSC senescence serves a protective role in the liver, with senescent HSCs downregulating ECM production and upregulating matrix-degrading enzymes. Genetic disruption of senescence pathways leads to the accumulation of proliferating HSCs and dramatically worsens fibrosis.^44^ A potential area of future investigation could be into the mechanism of GADD45A regulation of p21. Published data show that GADD45A and p21 directly interact and compete with some common binding targets, such as proliferating cell nuclear antigen (PCNA).^45^^,^^46^ As p21 and GADD45A have some overlapping functions in the cell cycle, it is possible that the coordination of their functions is tightly regulated during fibrogenesis.

### Therapeutic implications of targeting the UPR in liver fibrosis

Each arm of the UPR clearly influences HSC activation and fibrogenesis, in addition to the inter-regulation of these arms during stress. Our work shows that stable PERK deletion limited IRE1α and ATF6α signaling. This could be a consequence of long-term PERK deletion, but also highlights the crosstalk that occurs between the UPR pathways, and prior studies revealed that dual knockout of IRE1α and ATF6α from HSCs in vitro exacerbated fibrosis. Thus, targeting one or more of the UPR signaling pathways needs to be carefully investigated to determine the potential efficacy of such a strategy. Alleviation of ER stress through chemical chaperones has shown promise in vitro and in vivo. Administration of the chemical chaperone 4-phenylbutyric acid (4-PBA) limited CCl_4_-mediated fibrosis in mice, while limiting induction of the UPR and UPR-associated genes in LX-2 cells in vitro.^7^^,^^47^ The use of chemical chaperones therapeutically has potential, with some chaperones already FDA approved, and 4-PBA tested in clinical trials for different diseases.^48^^,^^49^ As for specific targeting of PERK, PERK inhibitors show promise for use in treating autoimmune diabetes, neurological disorders, and as an anti-cancer therapy.^50^ Analysis of PERK inhibitors on liver injury and fibrogenesis in vivo in future studies would provide critical insight into the therapeutic potential of these drugs in treating patients with chronic liver disease.

## CONCLUSION

In summary, our findings establish PERK as a critical regulator of TGFβ-induced HSC activation, with GADD45A serving as an important downstream effector. Identification of GADD45A binding partners and their regulation, as well as how canonical and non-canonical PERK signaling under pathological conditions facilitates HSC activation and fibrogenesis, will unveil novel and targetable fibrogenic mechanisms.

## Supplementary Material
