# Supplementary material for: The PERK–GADD45A axis is a key driver of hepatic stellate cell activation
Source: Hepatol Commun. 2026 Jun 19;10(7):e0980. doi: 10.1097/HC9.0000000000000980 (PMC13286415; doi:10.1097/HC9.0000000000000980)
Supplement: Supplementary file 1 [file hc9-10-e0980-s001.docx]

**The PERK-GADD45A axis is a key driver of hepatic stellate cell activation**

Barupala N and Misra J et al.

**Supplemental Methods**

**Immunoblot analyses**

Cells were lysed in 1% SDS supplemented with 1X Halt protease and phosphatase inhibitor cocktail (Thermo Scientific, #1861281). The lysates were boiled for 5 minutes, sonicated with a Branson Sonifier, and cleared by centrifugation at 12,000 × g. Protein concentrations were determined using the DC protein assay kit (Biorad, #5000112) with BSA as the standard. Equivalent amounts of protein were resolved by SDS-PAGE and transferred onto 0.2 μm nitrocellulose membranes and 0.2 or 0.4mm PVDF membrane via the Trans-Blot Turbo RTA nitrocellulose transfer system (BioRad, #1704270) and Mini-PROTEAN Tetra system (BioRad # BioRad, #1658004) respectively. The target proteins used in the immunoblot analyses, along with their primary antibodies, vendor details, and experimental dilutions, include p-PERK 1:1000 (Custom made, #LLY-71) (1), total PERK (CST, #C33E10), P-eIF2α (S51) 1:1000 (Abcam, #32157), total eIF2α 1:1000 (Cell Signaling, #5342), ATF4 antibody was prepared against the corresponding recombinant human proteins, which were affinity purified(2), Collagen I 1:1000 (Southern Biotech, #1310-01), αSMA 1:1000 (Abcam, #ab5694), Fibronectin 1:1000 (Abcam, #ab18265), GAPDH 1:3000 (Invitrogen, #2597762), Actin 1:5000 (Sigma, #A5441), HSC70 1:3000 (Santa Cruz, #sc-7298), and GADD45A 1:1000 (CST, #D17E8), SMAD2/3 1:1000 (Cell Signaling 3102S), P-SMAD3 1:1000 (Cell Signaling #9520S). After incubation with primary antibodies, the membranes were washed three times for 10 minutes each with TBS-T buffer, followed by incubation with secondary antibodies at room temperature with 1:5000 goat anti rabbit-HRP (Biorad, #170-6515), or goat anti-mouse-HRP (Biorad, #170-6516) or donkey anti goat (Invitrogen, #A15999) for 1h. The membranes were then washed three more times with TBS-T buffer for 10 minutes each. Immunoblot signals were visualized with Clarity Western ECL Substrate (Biorad, 170-5060) or SuperSignal West Femto Maximum Sensitivity Substrate (Thermo Scientific, #34094); images were captured using a Chemidoc MP imaging system (Biorad).

**Quantitative Real Time PCR (qRT-PCR) analysis**

Total RNA was extracted from cultured cells using Quick RNA MiniPrep Plus kit (Zymo research, #R1057) and quantified with a Nanodrop Spectrophotometer (Thermo Fisher Scientific). 1mg of total RNA was reverse transcribed into cDNA using the High-Capacity cDNA Reverse Transcription Kit (Thermo Fisher Scientific, #4368813) according to the manufacturer's instructions. After cDNA synthesis, samples were diluted fivefold in molecular biology-grade water, and PCR amplification was performed using PowerUp SYBR Green Master Mix (Thermo Fisher Scientific, #A25742) on an Applied Biosystems QuantStudio5 PCR system. Primers were used to quantify GADD45A, ACTA2, COL1A1, and CHOP transcript levels. The relative abundance of each transcript was determined using the ΔΔCT method, with GAPDH serving as an internal control, and data were normalized to the control group. Primers sequences used are listed below. *GADD45A* primers are purchased from Qiagen (Human-PPH00148B, Mouse-PPM02927C)

Human primers

| **Gene** | **Forward Primer (5’ to 3’)** | **Reverse Primer (5’ to 3’)** |
| --- | --- | --- |
| *ACTA2* | AAT GCA GAA GGA GAT CAC GG | TCC TGT TTG CTG ATC CAC ATC |
| *COL1A1* | TGT GAG GCC ACG CAT GAG | CAG ATC ACG TCA TCG CAC AA |
| *CHOP* | CAC TCT TGA CCC TGC TTC TC | CCA CTC TGT TTC CGT TTC CT |
| *GAPDH* | CTC TGC TCC TCC TGT TCG AC | TTA AAA GCA GCC CTG GTG GTG AC |
| *PERK* | ACG ATG AGA CAG AGT TGC GAC | ATC CAA GGC AGC AAT TCT CCC |
| *CDKN1A* | TAC CCT TGT GCC TCG CTC A | GGT AGA AAT CTG TCA TGC TGG TC |

Mouse primers

| **Gene** | **Forward Primer (5’ to 3’)** | **Reverse Primer (5’ to 3’)** |
| --- | --- | --- |
| *Acta2* | CAA CTG GTA TTG TGC TGG ACT C | AAG TCC AGA CGC ATG ATG G |
| *Col1a1* | CCA AAG GTC CTC GTG GTG | GGT CCA GCA TTT CCA GAG G |
| *Cdkn1a* | TTG TCG CTG TCT TGC ACT CT | AAT CTG TCA GGC TGG TCT GC |
| *Cdkn2a* | TGG ACC AGG TGA TGA TGA TG | GGA GAA GGT AGT GGG GTC CT |
| *Fn1* | GTG GCT GCC TTC AAT TCT C | GTG GGT TGC AAA CCT TCA AT |
| *Gadd45a* | GCT GCC AAG CTG CTC AAC | TCG TCG TCT TCG TCA GCA GCA |
| *Ki67* | TTG ACC GCT CCT TTA GGT ATG | CCT TGA TGG TTC CTT TCC AA |
| *Pcna* | GAA GAG GAG GCG GTA ACC AT | GGA GAC AGT GGA GTG GCT TT |
| *Tgfb1* | TTG CCC TCT ACA ACC AAC ACA A | GGC TTG CGA CCC ACG TAG TA |

**Polysome profiling**

LX-2 cells were treated with 5 ng/ml TGFβ or a vehicle control for 18 hours. Cycloheximide was added to each culture dish at a final concentration of 50 μg/ml, 10 minutes prior to harvesting. The cells were then rinsed with ice-cold PBS containing 50 μg/ml cycloheximide and lysed in 500μl of cold lysis buffer (20 mM Tris-HCl, pH 7.5, 100 mM NaCl, 10 mM MgCl2, 0.4% NP-40, and 50 μg/ml cycloheximide). After centrifugation at 15,871 × g for 10 minutes at 4°C, the cell lysates were layered onto 10–50% sucrose gradients and ultracentrifuged at 40,000 rpm for 2h at 4°C using a Beckman SW41Ti rotor. Polysome profiles of whole-cell lysates were then generated using a piston gradient fractionator (BioComp) and monitored at 254 nm with DataQuest Software, following established protocols ([20-22](#_ENREF_20)).

**Cell growth assay**

A total of 25,000 shNT, shPERK, or LX-2 cells were seeded onto individual wells of a 6-well cell culture plate, designated as day 0. Cells were allowed to grow, and cell numbers were measured as indicated. For GSK2656127 treatment, fresh inhibitor or vehicle control was added every other day. Two counts per condition were performed for each timepoint and averaged for each biological replicate.

**siRNA Knockdown**

LX-2 cells were transfected with 6 µL of siRNA using Lipofectamine RNAiMax (Thermo Fisher, #13778075) following the manufacturer’s instructions. After 24h, the cells were treated with TGFβ for the specified duration. The siRNAs purchased from Sigma Aldrich were Human siGADD45A (SASI_Hs01_00030953), Scramble siRNA (siRNA SIC001), siPERK (J-004883-12, Dharmacon), and siATF4 (J-005125-13, Dharmacon).

**Immunofluorescence**

Cells were seeded onto fibronectin-coated glass chamber slides, treated for 48h as described, rinsed with PBS, and fixed with 4% paraformaldehyde (PFA) in PBS for 20 minutes. Cells were permeabilized in 0.2% Triton-X-100 in PBS or left unpermeablized for extracellular deposition analysis. Cells were blocked in 3% BSA in PBS for 1 hour at room temperature before being incubated with primary antibodies (diluted between 1:200 and 1:50 in 3% BSA in PBS) overnight. After washing, samples were incubated with fluorescent secondary antibodies (diluted 1:250 to 1:125 in 3% BSA in PBS) for another hour. DAPI was applied for 5 minutes, after which the coverslips were mounted on slides. Imaging was performed using both an EVOS M5000 microscope (Invitrogen) and a Leica TCS SP8 Resonant-scanning confocal/multiphoton microscope, with 20x magnification for collagen deposition. Image analysis was performed using ImageJ. Collagen I was stained for collagen I (1:100, Southern Biotech, #1310-01) and nuclei were stained with DAPI.

For tissue staining, optimal cutting temperature (OCT) compound–embedded liver tissues were sectioned at 5 μm; 5-10 fields per tissue section were analyzed after immunofluorescent staining for semi-quantification, described as follows. The sections were fixed in 4% PFA for 30 minutes, permeabilized with 0.5% Triton X-100, and washed. Tissues were blocked with 10% Donkey serum for 1 hour at room temperature, then incubated with primary and secondary antibodies according to the manufacturer’s recommendations. ImageJ was used to quantify the integrated density of each image, and the JaCoP plugin was used to quantify colocalization. Imaging was performed using a Zeiss AxioObserver Z1 microscope with the same laser intensity and power within each experiment.

**Mouse HSC Isolation**

Primary mouse hepatic stellate cells (mHSCs) were isolated from either wild-type c57BL/6, *Gadd45a*^fl/fl^, and *Gadd45a*^fl/fl^*Pdgfrb*^CreERT2^ mice by cannulating the portal vein, perfusing the liver with pronase and collagenase, and then using density-gradient centrifugation to isolate the HSCs, following established protocols ([13](#_ENREF_13)). The isolated cells were then infected with adenoviruses expressing either Cre-recombinase (Ad5CMVCre-eGFP; AdCre) or a control LacZ (Ad5CMVcytoLacZ-GFP; LacZ), both sourced from the University of Iowa Viral Vector Core. After 24h of infection, the mHSCs were serum-starved for 8 hours before being treated with either 2 ng/mL mTGFβ or a vehicle control for another 24h.

**Immunohistochemical Analyses for tissues**

Immunohistochemistry (IHC) was performed on mice livers sectioned at (4-6 mm). Briefly, after deparaffinizing the antigen, unmasking was done using citrate-based antigen unmasking solution (H3401-Vector Laboratory). Liver sections were incubated with specific antibodies against collagen 1 (1:200 dilution) at 4°C overnight. Reactions were detected with the Vector Laboratories DAB Kit (Burlingame, CA, USA) according to the manufacturer's protocol. The slides were scanned at x20 magnification using a digital scanner (Aperio Scanscope CS System, Aperio Digital Pathology, Leica Biosystems). Semi-quantification was done for all IHC stains in a blinded fashion by taking 6-8 non-overlapping images from each tissue section from at least 3 mice per group using ImageScope version 12.3.3 (Leica Biosystems) with the positive pixel count v9 algorithm. Collagen deposition was evaluated in paraffin-embedded liver sections (4-6 μm) stained with Sirius Red (IHC World- Cat# IW-3012). The percentage area of Sirius red positivity was quantified using ImageJ and presented in the adjacent graph.

For immunofluorescent (IF) staining of tissues, optimal cutting temperature (OCT)–embedded liver tissues were sectioned at 5 μm; 5-10 fields per tissue section were analyzed after immunofluorescent staining for semi-quantification as described below. Immunofluorescence for Desmin (Life Technologies, MA513259; 1:25) was performed in frozen liver sections co-stained with Collagen 1 (Southern Biotech 1301-01; 1:100) and p21 (CST 2947S; 1:200). Stains were visualized using a confocal microscope (Zeiss-Axio Observer.Z1), and the area of colocalization was quantified using ImageJ JACoP using the Pearson correlation coefficient.

**References**

1. Tenkerian C, Krishnamoorthy J, Mounir Z, Kazimierczak U, Khoutorsky A, Staschke KA, Kristof AS, et al. mTORC2 Balances AKT Activation and eIF2α Serine 51 Phosphorylation to Promote Survival under Stress. Molecular Cancer Research 2015;13:1377-1388.

2. Fusakio ME, Willy JA, Wang Y, Mirek ET, Al Baghdadi RJ, Adams CM, Anthony TG, et al. Transcription factor ATF4 directs basal and stress-induced gene expression in the unfolded protein response and cholesterol metabolism in the liver. Mol Biol Cell 2016;27:1536-1551.
