# Supplementary material for: The PERK–GADD45A axis is a key driver of hepatic stellate cell activation
Source: Hepatol Commun. 2026 Jun 19;10(7):e0980. doi: 10.1097/HC9.0000000000000980 (PMC13286415; doi:10.1097/HC9.0000000000000980)
Supplement: Supplementary file 2 [file hc9-10-e0980-s002.pdf]

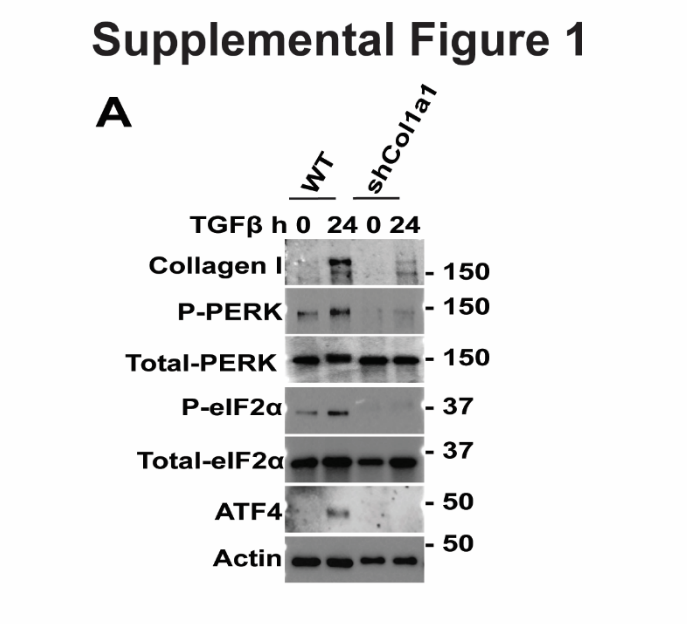

Supplemental Figure 1. Collagen I expression drives PERK signaling. LX-2 cells expressing shRNA targeting *COL1A1* or a non-targeting control (shNT) were treated with TGFβ (5ng/mL) for 0 and 24h. Lysates were harvested and immunoblotted with the indicated antibodies.
