# Supplementary material for: The PERK–GADD45A axis is a key driver of hepatic stellate cell activation
Source: Hepatol Commun. 2026 Jun 19;10(7):e0980. doi: 10.1097/HC9.0000000000000980 (PMC13286415; doi:10.1097/HC9.0000000000000980)
Supplement: Supplementary file 3 [file hc9-10-e0980-s003.pdf]

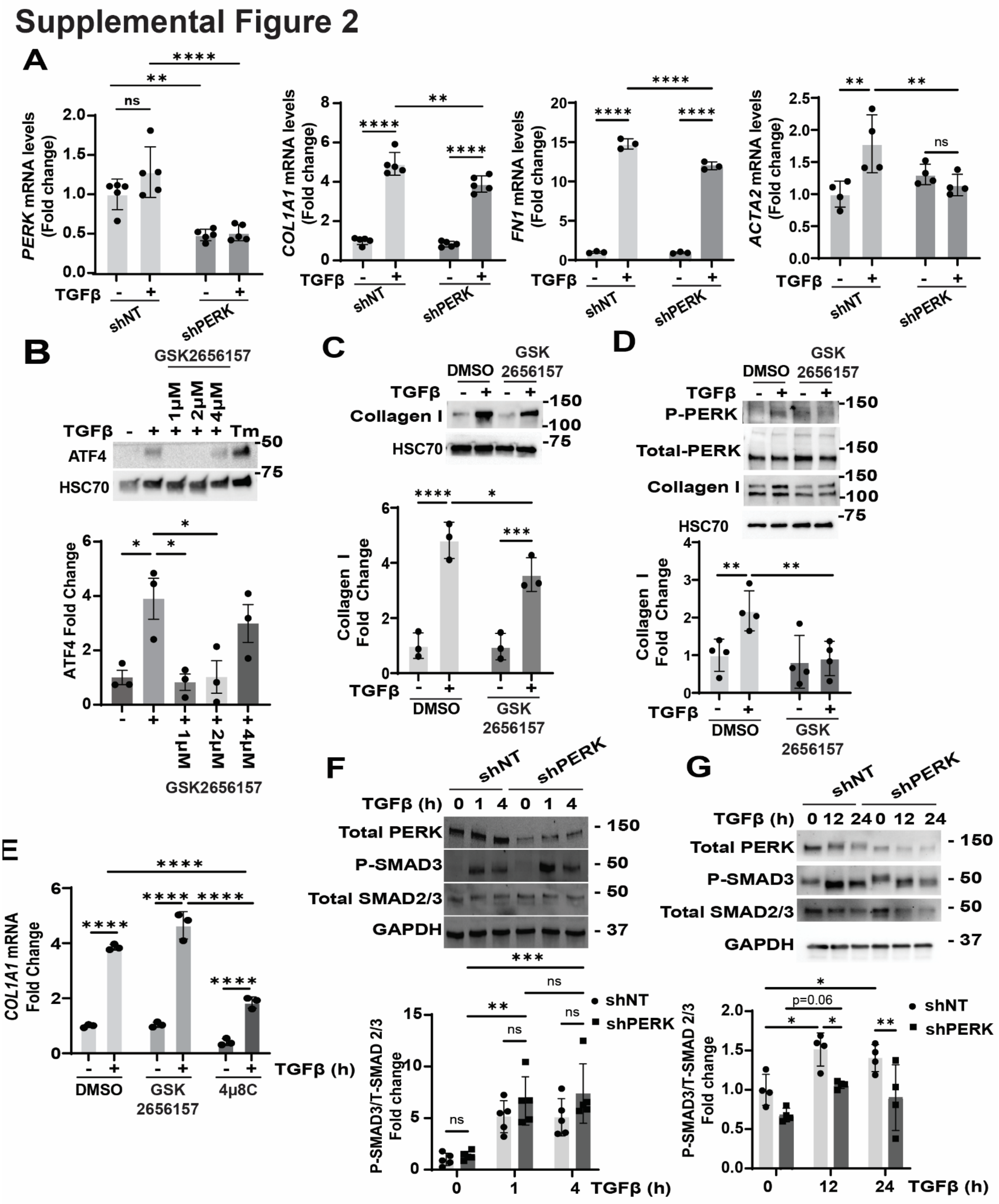

Supplemental Figure 2. PERK loss or inhibition limits HSC activation. (A) shPERK and shNT cells were treated with TGFβ (5ng/mL) for 24h. followed by qPCR analyses of *PERK*, *COL1A1*, *FN1*, and *ACTA2*. (n=3-5). (B) LX-2 cells were pretreated with GSK2656157 (1, 2, or 4μM) followed by treatment with TGFβ for 24h. Cell lysates

were harvested and analyzed by immunoblot (N=3). Tunicamycin treatment served as a positive control. (C and D). LX-2 cells (C) and isolated mouse HSC cells (D) were pre-treated with GSK2656157 (2μM) followed by treatment with TGFβ for 24h. Cell lysates were harvested and analyzed by immunoblot (N=3-4). (E). LX-2 cells were pretreated with GSK2656157 (2μM) or 4μ8C (15mM) followed by TGFβ (5ng/mL) for 24h. *COL1A1* levels were analyzed by qPCR (N=3). (F and G). shNT or shPERK cells were treated with TGFβ (5ng/mL) for the indicated time points and analyzed by immunoblotting. (N=5 for F, N=4 for G). Statistical significance was denoted by *; * = $p < 0.05$, ** = $p < 0.01$, *** = $p < 0.001$, and ****= $p < 0.0001$ by Two-way ANOVA (A, C-F), or One-way ANOVA (B). Error bars indicate mean ± SD.
