# Supplementary material for: The PERK–GADD45A axis is a key driver of hepatic stellate cell activation
Source: Hepatol Commun. 2026 Jun 19;10(7):e0980. doi: 10.1097/HC9.0000000000000980 (PMC13286415; doi:10.1097/HC9.0000000000000980)
Supplement: Supplementary file 4 [file hc9-10-e0980-s004.pdf]

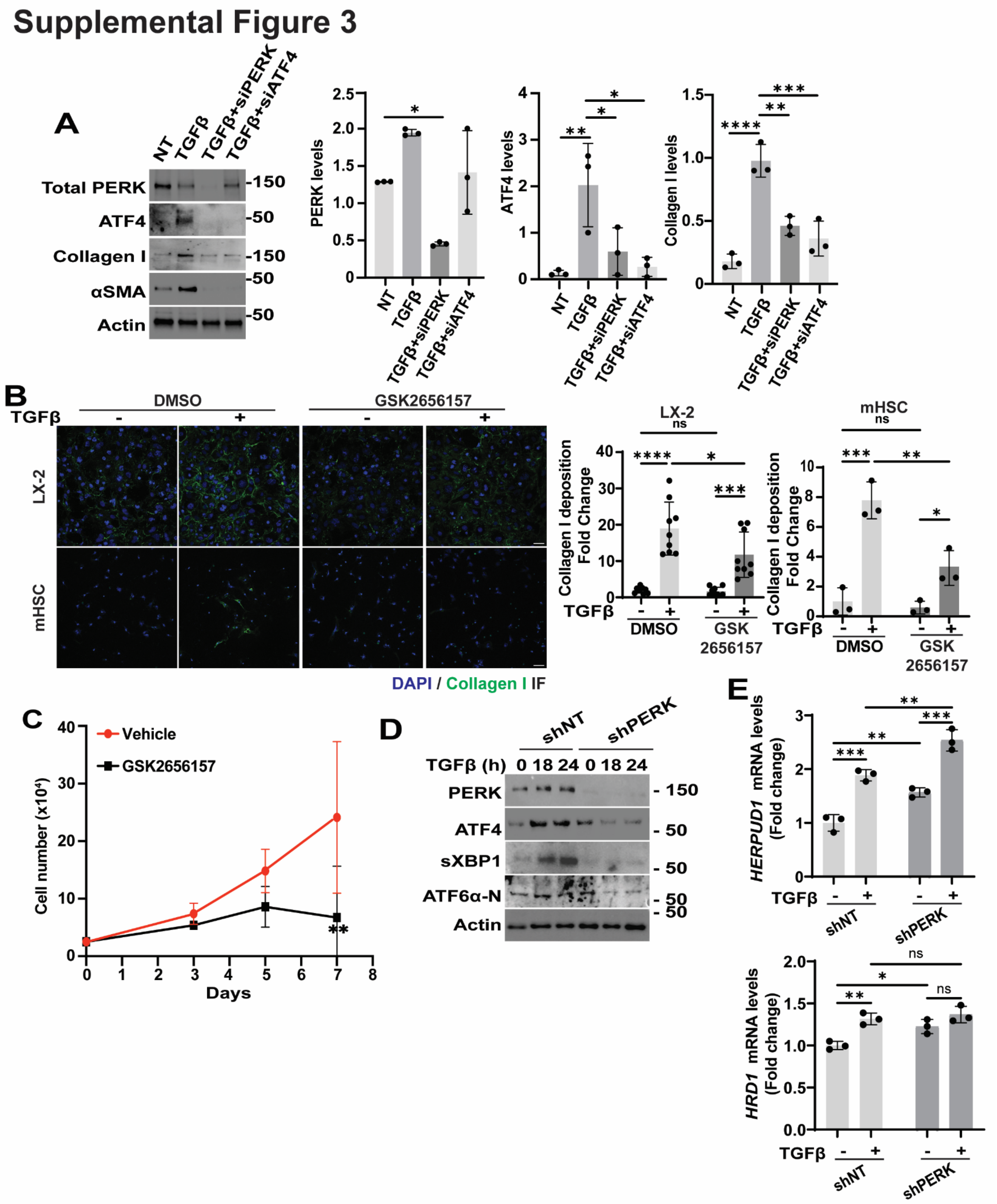

Supplemental Figure 3. PERK disruption limits HSC activation, proliferation, and disrupts UPR signaling. (A) LX-2 cells were transfected with siRNA targeting PERK or ATF4; 24h. post transfection cells were treated with TGFβ (5ng/mL) for 24h, followed by immunoblot analyses with the indicated antibodies (n=3). (B) LX-2 cells and

isolated mouse HSC cells were pre-treated with GSK2656157 (2mM) followed by treatment with TGFβ for 48h. Cells were then permeabilized and immunostained for collagen I (green) (n=3, SB = 50μm). (C) LX-2 cells were seeded and treated with 1 μM GSK2656157 for every 24 hour counted over 7 days to assess cell proliferation (n=3). (D) shNT and shPERK LX-2 cells were treated with TGFβ for different time points as indicated followed by immunoblot analyses with the indicated antibodies. (E) shNT and shPERK LX-2 cells were treated with TGFβ for 24h followed by qPCR analyses of the indicated mRNAs (n=3). Statistical significance was denoted by *; * = $p < 0.05$, ** = $p < 0.01$, *** = $p < 0.001$, and ****= $p < 0.0001$ by One-way ANOVA (A) or Two-way ANOVA (B-E). Error bars indicate mean ± SD.
