# Supplementary material for: The PERK–GADD45A axis is a key driver of hepatic stellate cell activation
Source: Hepatol Commun. 2026 Jun 19;10(7):e0980. doi: 10.1097/HC9.0000000000000980 (PMC13286415; doi:10.1097/HC9.0000000000000980)
Supplement: Supplementary file 5 [file hc9-10-e0980-s005.pdf]

Supplemental Figure 4

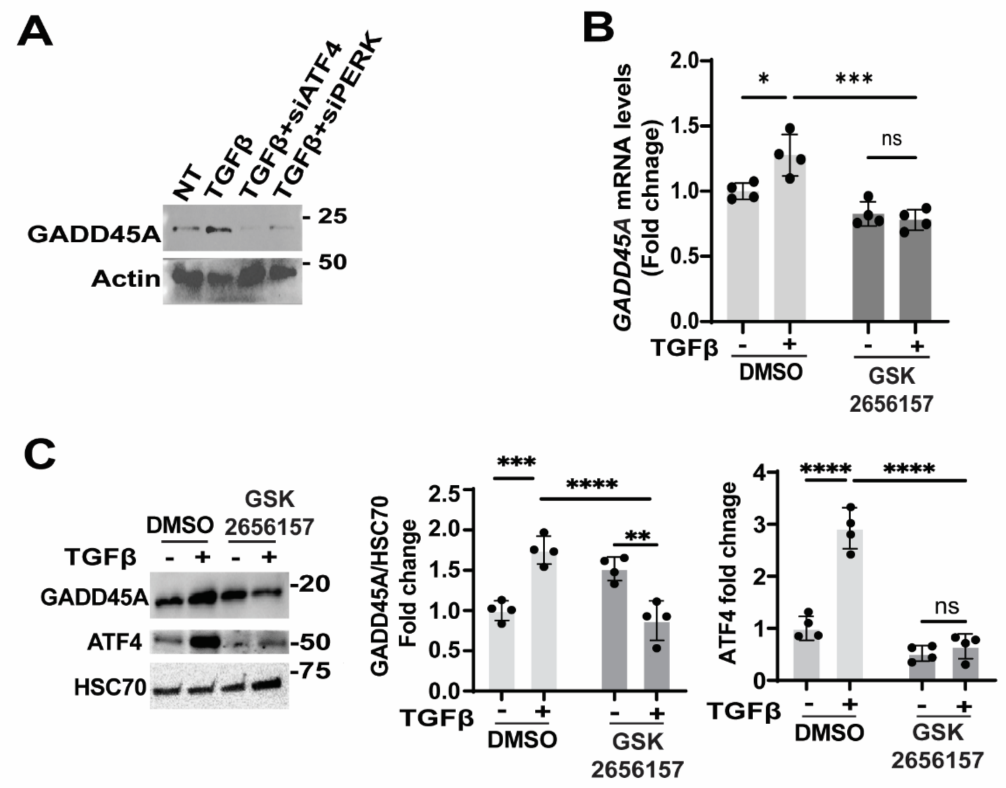

Supplemental Figure 4. GADD45A is regulated by PERK signaling in response to TGFβ. (A) LX-2 cells were transfected with siRNA targeting PERK or ATF4; 24h. post transfection cells were treated with TGFβ (5ng/mL) for 24h. Immunoblot analyses of GADD45A was performed. (B and C). LX-2 cells were pretreated with GSK2656157 (2mM) followed by treatment with TGFβ for 24h. qPCR analysis (C) or immunoblotting were performed to analyze levels of GADD45A and ATF4 (N=4). Statistical significance was denoted by *; * = $p < 0.05$, *** = $p<0.001$, and ****=$p<0.0001$ by Two-way ANOVA (C and D). Error bars indicate mean ± SD; (n=3 if not noted otherwise).
