# Supplementary material for: The PERK–GADD45A axis is a key driver of hepatic stellate cell activation
Source: Hepatol Commun. 2026 Jun 19;10(7):e0980. doi: 10.1097/HC9.0000000000000980 (PMC13286415; doi:10.1097/HC9.0000000000000980)
Supplement: Supplementary file 6 [file hc9-10-e0980-s006.pdf]

Supplemental Figure 5

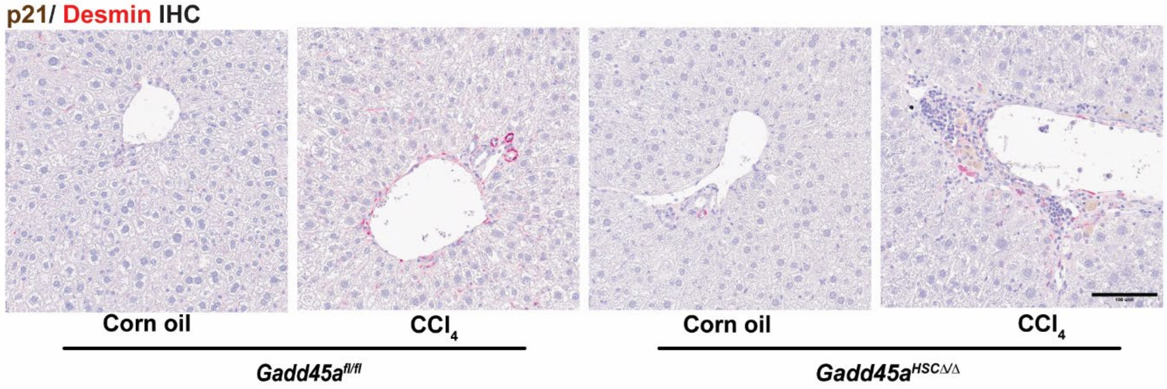

Supplemental Figure 5. *Gadd45a* loss enhanced p21 expression in response to CCl_4 treatment. Immunostaining of p21 (brown) and Desmin (red) in *Gadd45a*$^{fl/f}$ and *Gadd45a*$^{HSC\Delta/\Delta}$ mice treated with ether CO or CCl_4.
