# Supplementary material for: The PERK–GADD45A axis is a key driver of hepatic stellate cell activation
Source: Hepatol Commun. 2026 Jun 19;10(7):e0980. doi: 10.1097/HC9.0000000000000980 (PMC13286415; doi:10.1097/HC9.0000000000000980)
Supplement: Supplementary file 7 [file hc9-10-e0980-s007.pdf]

# Supplemental Figure 6

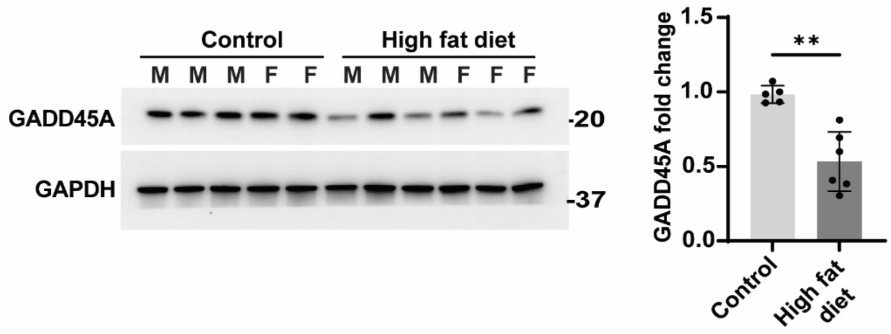


**Supplemental Figure 6.** Whole liver lysates from mice fed a high fat diet for 8 weeks or matched control diet were immunoblotted for GADD45A (n=5 and 6 per group) analyzed by T-test. Statistical significance was denoted by ** = p< 0.01. Error bars indicate mean ± SD.
